# Post-diagnosis hemoglobin change associates with overall survival of multiple malignancies – results from a 14-year hospital-based cohort of lung, breast, colorectal, and liver cancers

**DOI:** 10.1186/1471-2407-13-340

**Published:** 2013-07-10

**Authors:** Shaogui Wan, Yinzhi Lai, Ronald E Myers, Bingshan Li, Juan P Palazzo, Ashlie L Burkart, Guokai Chen, Jinliang Xing, Hushan Yang

**Affiliations:** 1 Division of Population Science, Department of Medical Oncology, Kimmel Cancer Center, Thomas Jefferson University, Philadelphia, PA, 19107, USA; 2 Department of Pathology, Thomas Jefferson University, Philadelphia, PA, 19107, USA; 3 Center for Human Genetics Research, Department of Molecular Physiology & Biophysics, Vanderbilt University, Nashville, TN, 37232, USA; 4 Center for Molecular Medicine, National Heart Blood Lung Institute, National Institutes of Health, Bethesda, MD, 20892, USA; 5 State Key Laboratory of Cancer Biology, Cell Engineering Research Centre & Department of Cell Biology, Fourth Military Medical University, Xi’an, 710032, China

**Keywords:** Hemoglobin, Survival, Prognosis

## Abstract

**Background:**

Anemia refers to low hemoglobin (Hb) level and is a risk factor of cancer patient survival. The National Comprehensive Cancer Network recently suggested that post-diagnosis Hb change, regardless of baseline Hb level, indicates the potential presence of anemia. However, there is no epidemiological study evaluating whether Hb change has direct prognostic values for cancer patients at the population level.

**Methods:**

We identified 6675 patients with a diagnosis of primary lung, breast, colorectal, or liver cancer who visited the Kimmel Cancer Center at the Thomas Jefferson University from 1998 to 2011. All patients had at least two Hb measurements within the first six months after diagnosis. We analyzed the main, dose-dependent, and time-dependent effects of Hb changes on patient survival.

**Results:**

Compared to patients with a low Hb change (|∆Hb|≤2.6), those having a |∆Hb|>2.6 exhibited a significantly shorter survival (hazard ratio=1.40, 95% confidence interval 1.31-1.50, *P*=4.5 × 10^-22^, *P*_log rank_=1.6 × 10^-39^). This association remained significant across the four cancer types. Bootstrap resampling validated these findings 100% of the time with *P*<0.01 in all patients and in patients of individual cancers. The association exhibited an apparent U-shape dose-dependent pattern. Time-dependent modeling demonstrated that the effect of Hb change on the survival of the overall patient population persisted for approximately 4.5 years after diagnosis.

**Conclusion:**

Post-diagnosis Hb change associates with the survival of multiple cancers and may have clinical values in tailoring anti-anemia treatments. Because Hb level is frequently measured during cancer treatment, Hb changes may be a potentially important variable in building cancer prognosis models.

## Background

Anemia is a condition that develops when whole blood lacks sufficient healthy red blood cells or hemoglobin (Hb), an oxygen-carrying protein within red blood cells. Cancer-associated anemia is one of the most common paraneoplastic syndromes during cancer progression or treatment and is prevalent in 30% to 90% of cancer patients [1]. Although anemia incidence varies with cancer types, stages and patient characteristics, it has been estimated that over 40% of all cancer patients are anemic at diagnosis, a rate that increases by an additional 40% after chemotherapy or radiation therapy treatments [1-4]. Because cancer-associated anemia has been documented as an adverse prognostic factor as well as predictor of treatment response, [5,6] evaluations of anemia and anti-anemia treatments have significant clinical implications.

The diagnosis and treatment of anemia are influenced by various factors, such as hemorrhage, hemolysis, nutritional deficiency, hereditary disease, renal dysfunction, and systemic chemotherapy or radiation therapy [7,8]. Since clinical symptoms of anemia start slowly, Hb level is currently the most important predictor in guiding anemia evaluation and treatment, regardless of the underlying causes [3]. Correction of cancer-associated anemia is usually achieved by blood transfusion with packed red blood cells (PRBCs) or erythropoiesis-stimulating agents (ESAs). However, considerable controversy regarding the safety and restrictions on the correction modality of cancer-associated anemia has been recently reported. For instance, several recent meta-analysis studies reported that ESA administrations, while reducing the incidence of clinically defined anemia, may confer an adverse survival to patients exhibiting a large change in Hb levels during treatment [9-12]. In comparison, other studies did not identify such significant effects of ESA use on patient mortality [13,14]. These studies suggested the complexity in the diagnosis and tailored treatment of anemia in the context of broad variations in Hb levels.

Given the wide variations in Hb level among cancer patients and even healthy individuals, it is impractical to define a clinically universal “normal” Hb value. Moreover, during anti-anemia treatment in clinics, it remains controversial as to the time to start initial treatment and the targeted range of Hb levels. For example, the baseline Hb values that trigger ESA treatment for patients with cancer- or therapy-induced anemia range from 8 to 11 g/dL under different practice guidelines [3,15-18]. The newly updated National Comprehensive Cancer Network (NCCN) guideline suggests that in non-anemic patients with a high baseline Hb level, a drop of 2 g/dL or more should be evaluated for the presence of anemia [3]. This raises the question whether a change in Hb levels during follow-up or treatment, regardless of baseline or single time point Hb levels, can directly predict patient prognosis. In the present study, we sought to utilize a large hospital-based cancer patient cohort to comprehensively evaluate post-diagnosis Hb change (∆Hb) as a predictor of overall survival in patients with lung, breast, colorectal, or liver cancer, four of the most common causes of cancer-related deaths. To the best of our knowledge, this is the first population-based epidemiological study that evaluates Hb changes (either an increase or a decrease) in association with cancer patient prognosis.

## Methods

### Study population

Subjects in this study were identified from a hospital-based cohort of patients with histologically confirmed primary lung, breast, colorectal, or liver cancer, who visited the Kimmel Cancer Center at Thomas Jefferson University with an initial diagnosis date from 1998 to 2011. For this analysis, a total of 6675 patients were included who had at least two Hb measurements within the first six months after primary cancer diagnosis. This study was approved by the Institutional Review Board of Thomas Jefferson University.

### Data collection

Demographic and clinical data were obtained from medical chart review. Demographic variables included age, gender, and ethnicity. Clinical variables included tumor stage, tumor grade and treatments including surgery, chemotherapy and radiation therapy. The routine clinical laboratory tests of complete blood count panel, including Hb values and dates of measurement, were obtained from systemic review of electronic medical charts. The maximum and minimum values of Hb levels within six months after cancer diagnosis were used to calculate Hb change (∆Hb), and the date of Hb measurements were used to determine the plus or minus sign of ∆Hb. When the minimum value was measured before the maximum value, the sign of ∆Hb was plus, indicating an increase in Hb level. Otherwise, the sign of ∆Hb was minus, indicating a decrease in Hb level.

### Statistical analysis

The clinical endpoint analyzed in this study was overall survival of cancer patients. Overall survival time was defined as the time from initial cancer diagnosis to death from any cause or last follow-up. Patients who were alive at last follow-up were censored for analysis. The associations between ∆Hb and overall survival were estimated using hazard ratio (HR) and 95% confidence interval (95% CI) calculated by multivariate Cox proportional hazards model, adjusting for age, gender, ethnicity, tumor stage, tumor grade, surgery, chemotherapy, and radiation therapy, where appropriate. The results of the main effects analyses were internally validated using the bootstrap resampling method [19]. A total of 100 bootstrap samples were generated for each analysis. Each time, a bootstrap sample was drawn from the original dataset and the *P* value for the analysis was calculated. The number of times with a *P* value < 0.01 was counted. Dose-dependent analyses were conducted, assuming ∆Hb as both a categorical and a continuous variable, by multivariate Cox proportional hazards model and fractional polynomial regression model, respectively, adjusting for age, gender, ethnicity, tumor stage, tumor grade, surgery, chemotherapy, and radiation therapy [20]. The distributions of absolutely changed Hb values between gender and cancer sites were compared by Student’s *t* test. Time-dependent analyses were conducted using flexible parametric modeling framework that analyzes the interaction between ∆Hb and survival time, and confers a time-dependent effect of ∆Hb on patient survival adjusting for other host and clinical variables [21]. Survival curves were constructed using the Kaplan-Meier method and compared using the log rank test with anemia indicated by a Hb level of less than 12 g/dL [3]. Statistical analyses in this study were conducted using SAS 9.2 (SAS Institute, Cary, NC) and STATA 12.0 (STATA Corp., College Station, TX) software packages. All *P* values were 2-sided. *P*≤0.05 was considered statistical significant.

## Results

### Characteristics of study population

**Figure 1:**
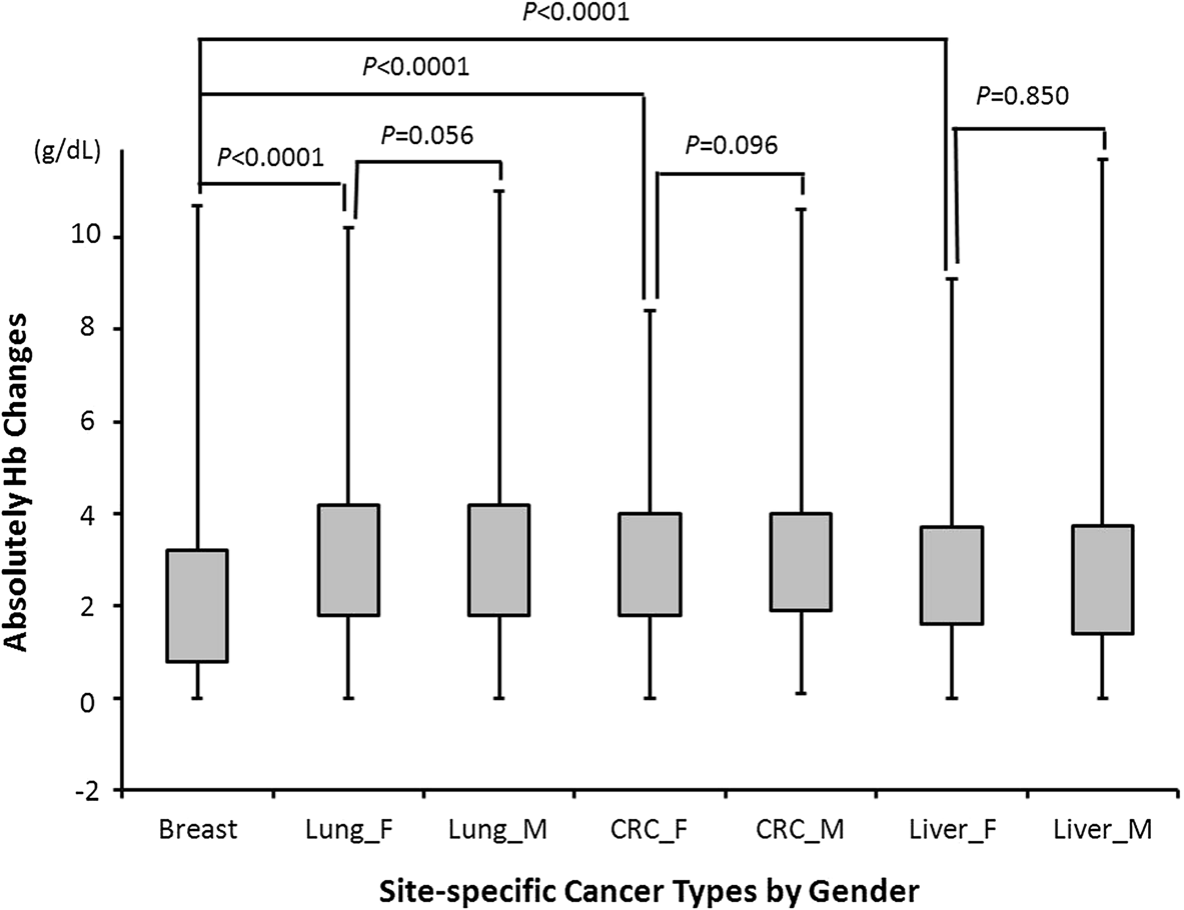
**Hb changes in different cancer types by gender.** Breast, breast cancer patients, Lung_F, female lung cancer patients; Lung_M, male lung cancer patients; CRC_F, female colorectal cancer patients; CRC_M, male colorectal cancer patients; Liver_F, female liver cancer patients; Liver_M, male liver cancer patients. The grey boxes were quartile range of absolutely Hb changes from 25-75%, the upper and lower error bars indicated maximum and minimum changed values of Hb, respectively. The indicated groups were compared by Student’s *t* test.

A total of 6675 cancer patients were included in the analysis for this study, with an average age of 62.4 (± standard deviation, 11.3) years. The distributions of host variables were summarized in Table 1. There were 2367 lung cancer patients, 1739 breast cancer patients, 1860 colorectal cancer (CRC) patients, and 709 liver cancer patients, with an average age of 65.5 (± 11.3), 56.3 (± 13.3), 64.7 (± 13.8), and 61.0 (± 11.3), respectively. In all cancer patients, a relatively similar distribution of patients was observed among stages 1 to 4 (from 18.08% to 26.43%). The majority of patients had moderately (33.86%) or poorly (22.68%) differentiated tumors. Most patients received surgical resection (72.39%), especially those with breast (94.54%) and colorectal cancers (88.71%). Approximately 43% of patients received chemotherapy with a relatively similar distribution across the four cancer types. About 28.9% of patients received radiation therapy. The median follow-up time of all patients in this study was 26.8 months (quartile range, 8.8-69.4) and the median survival time of censored patients was 58.7 months (quartile range, 26.2-100.4). Figure 1 shows the distribution of absolute ∆Hb (|∆Hb|) in different cancers by gender status. A borderline significant difference between male and female patients was noticed in lung (*P*=0.056) and colorectal (*P*=0.096), but not liver cancer patients (*P*=0.850). The |∆Hb| value in breast cancer patients was significantly lower than that of the female patients of the other three cancers with *P*<0.0001.

**Table 1:** Characteristics of the study population

	**All cancers (n=6675)**	**Lung (n=2367)**	**Breast (n=1739)**	**CRC (n=1860)**	**Liver (n=709)**
**Variables**	**No. of patients (%)**	**No. of patients (%)**	**No. of patients (%)**	**No. of patients (%)**	**No. of patients (%)**
**Age (Mean ± SD)**	62.4 ± 11.3	65.5 ± 11.3	56.3 ± 13.3	64.7 ± 13.8	61.0 ± 11.3
**Gender**					
Female	4081(61.14)	1232(52.05)	1739(100)	949(51.02)	161(22.71)
Male	2594(38.86)	1135(47.95)		911(48.98)	548(77.29)
**Ethnicity**					
Caucasian	4758(71.28)	1780(75.2)	1123(64.58)	1403(75.43)	452(63.75)
Black	1445(21.65)	468(19.77)	491(28.23)	359(19.3)	127(17.91)
Others	472(7.07)	119(5.03)	125(7.19)	98(5.28)	130(18.33)
**Tumor stages**					
Stage 0	325(4.87)	1(0.04)	264(15.18)	60(3.23)	
Stage 1	1764(26.43)	533(22.52)	605(34.79)	419(22.53)	207(29.2)
Stage 2	1272(19.06)	158(6.68)	527(30.3)	454(24.41)	133(18.76)
Stage 3	1207(18.08)	465(19.65)	164(9.43)	418(22.47)	160(22.57)
Stage 4	1659(24.85)	1042(44.02)	119(6.84)	385(20.7)	113(15.94)
Unknown	448(6.71)	168(7.09)	60(3.45)	124(6.67)	96(13.54)
**Tumor grades**					
Well	515(7.71)	124(5.24)	155(8.91)	172(9.25)	64(9.03)
Moderately	2260(33.86)	427(18.04)	575(33.06)	1138(61.18)	120(16.93)
Poorly	1514(22.68)	630(26.62)	605(34.79)	250(13.44)	29(4.09)
Not determined	2386(35.75)	1186(50.1)	404(23.23)	300(16.13)	496(69.96)
**Chemotherapy**					
No	2258(33.83)	258(10.9)	989(56.87)	918(49.35)	93(13.12)
Yes	2865(42.92)	948(40.05)	705(40.54)	840(45.16)	372(52.47)
Unknown	1552(23.25)	1161(49.05)	45(2.59)	102(5.48)	244(34.41)
**Radiation**					
No	2933(43.94)	208(8.79)	1097(63.08)	1492(80.22)	136(19.18)
Yes	1929(28.9)	970(40.98)	582(33.47)	335(18.01)	42(5.92)
Unknown	1813(27.16)	1189(50.23)	60(3.45)	33(1.77)	531(74.89)
**Surgery**					
No	1134(16.99)	615(25.98)	95(5.46)	210(11.29)	214(30.18)
Yes	4832(72.39)	1200(50.7)	1644(94.54)	1650(88.71)	338(47.67)
Unknown	709(10.62)	552(23.32)			157(22.14)
**Vital status**					
Alive	3133(46.94)	572(24.17)	1374(79.01)	986(53.01)	201(28.35)
Dead	3542(53.06)	1795(75.83)	365(20.99)	874(46.99)	508(71.65)

### Post-diagnosis Hb changes and the overall survival of cancer patients

The associations between ∆Hb and patient survival were estimated by multivariate Cox proportional hazard model. First, we analyzed |∆Hb| and overall survival in all cancer patients as well as in patients with individual cancers using a median cut-off value of 2.6 g/dL measured in all cancer patients. As shown in Table 2, patients with a higher level of |∆Hb| exhibited a significantly poorer survival, compared to those with a low |∆Hb| level, with an HR of 1.40 (95% CI 1.31-1.50, *P*=4.5×10^-22^, *P*_log rank_=1.6×10^-39^). Similar results were found in patients with either a decreased Hb (∆Hb<−2.6) (HR=1.35, 95% CI 1.25-1.46, *P*=3.0×10^-15^) or an increased Hb (∆Hb>2.6) (HR=1.53, 95%CI 1.39-1.69, *P*=5.7×10^-18^). The significant association between |∆Hb| and patient survival remained prominent across the four cancer types included in this study, with an HR (95% CI, *P* value, log rank *P* value) of 1.32 (1.20-1.46, *P*=2.7×10^-8^, *P*_log rank_=8.9×10^-4^), 1.49 (1.19-1.86, *P*=4.3×10^-4^, *P*_log rank_=6.5×10^-10^), 1.46 (1.27-1.68, *P*=7.9×10^-8^, *P*_log rank_=4.6×10^-8^), and 1.45 (1.20-1.74, *P*=7.9×10^-5^, *P*_log rank_=1.6×10^-2^) for lung, breast, colorectal, and liver cancer, respectively. Kaplan Meier analysis demonstrated a significantly different overall survival time between patients with higher- and lower-than-median |∆Hb| in all cancer patients as well as in patients with individual cancers (data not shown). We then conducted internal validation using bootstrap resampling and demonstrated that the significant association between |∆Hb| and patient survival was validated 100% of times in all cancer patients combined, as well as in patients with individual cancer types (Table 2). In concordance with previous reports, anemia at baseline (baseline Hb < 12 g/L) conferred a significant adverse effect on patient survival (Additional file 1: Table S1). A joint analysis between baseline Hb level and Hb change in the combined cohort and individual cancer sites indicated the Hb changes added additional predictive value compared to baseline Hb alone (Additional file 1: Table S2). For instance, in the overall cohort, compared to patients with high baseline Hb and small Hb change, the risk of death was 1.23 (95% CI 1.09-1.39), *P*=0.0008, 1.34 (95% CI 1.19-1.51), *P*=1.6×10^-6^, and 1.75 (95% CI 1.58-1.94), *P*=4.9×10^-26^) for those with a high baseline Hb and large Hb change, low baseline Hb and small Hb change, and low baseline Hb and large Hb change, respectively.

**Table 2:** The association between Hb changes and the overall survival of cancer patients

**Cancers**	**Hb changes**		**Dead**	**Alive**	HR (95% CI)*	***P *value**	**Validation†**	**Log-rank *P***
All cancers								
	By |∆Hb|							
		|∆Hb| ≤ 2.6	1585	1831	1.00			
		|∆Hb| > 2.6	1957	1302	1.40 (1.31-1.50)	4.5 x 10^-22^	100	1.6 x 10^-39^
	By ∆Hb							
		−2.6 ≤ ∆Hb ≤ 2.6	1585	1831	1.00			
		∆Hb < −2.6	1357	975	1.35 (1.25-1.46)	3.0 x 10^-15^	100	
		∆Hb > 2.6	600	327	1.53 (1.39-1.69)	5.7 x 10^-18^	100	9.4 x 10^-40^
Lung cancer								
	By |∆Hb|							
		|∆Hb| ≤ 2.6	756	281	1.00			
		|∆Hb| > 2.6	1039	291	1.32 (1.20-1.46)	2.7 x 10^-8^	100	8.9 x 10^-4^
	By ∆Hb							
		−2.6 ≤ ∆Hb ≤ 2.6	756	281	1.00			
		∆Hb < −2.6	774	246	1.25 (1.12-1.38)	3.7 x 10^-5^	97	
		∆Hb > 2.6	265	45	1.59 (1.38-1.84)	1.8 x 10^-10^	100	2.3 x 10^-5^
Breast cancer								
	By |∆Hb|							
		|∆Hb| ≤ 2.6	195	927	1.00			
		|∆Hb| > 2.6	170	447	1.49 (1.19-1.86)	4.3 x 10^-4^	100	6.5 x 10^-10^
	By ∆Hb							
		−2.6 ≤ ∆Hb ≤ 2.6	195	927	1.00			
		∆Hb < −2.6	135	377	1.45 (1.14-1.84)	0.002	70	
		∆Hb > 2.6	35	70	1.65 (1.14-2.40)	0.008	58	1.5 x 10^-9^
Colorectal cancer								
	By |∆Hb|							
		|∆Hb| ≤ 2.6	360	506	1.00			
		|∆Hb| > 2.6	514	480	1.46 (1.27-1.68)	7.9 x 10^-8^	100	4.6 x 10^-8^
	By ∆Hb							
		−2.6 ≤ ∆Hb ≤ 2.6	360	506	1.00			
		∆Hb < −2.6	281	299	1.46 (1.24-1.71)	3.4 x 10^-6^	100	
		∆Hb > 2.6	233	181	1.46 (1.23-1.73)	1.4 x 10^-5^	100	4.5 x 10^-8^
Liver cancer								
	By |∆Hb|							
		|∆Hb| ≤ 2.6	274	117	1.00			
		|∆Hb| > 2.6	234	84	1.45 (1.20-1.74)	7.9 x 10^-5^	100	0.016
	By ∆Hb							
		−2.6 ≤ ∆Hb ≤ 2.6	274	117	1.00			
		∆Hb < −2.6	167	53	1.50 (1.23-1.83)	8.0 x 10^-5^	87	
		∆Hb > 2.6	67	31	1.33 (1.01-1.76)	0.044	34	0.011

*Adjusted for age, gender, ethnicity, tumor stage, tumor grade, chemotherapy, radiation therapy, and surgery. †The number of times with *P*<0.01 in 100 resamplings of bootstrap internal validation. |∆Hb|, the maximum absolute Hb change during the first six months after cancer diagnosis. The cut off was determined using the median value (2.6 g/dL) of all cancer patients. ∆Hb<−2.6 indicated decreased Hb changes and ∆Hb>2.6 indicated increased Hb changes.

### Dose-dependent effects of Hb changes on cancer patient survival

**Figure 2:**
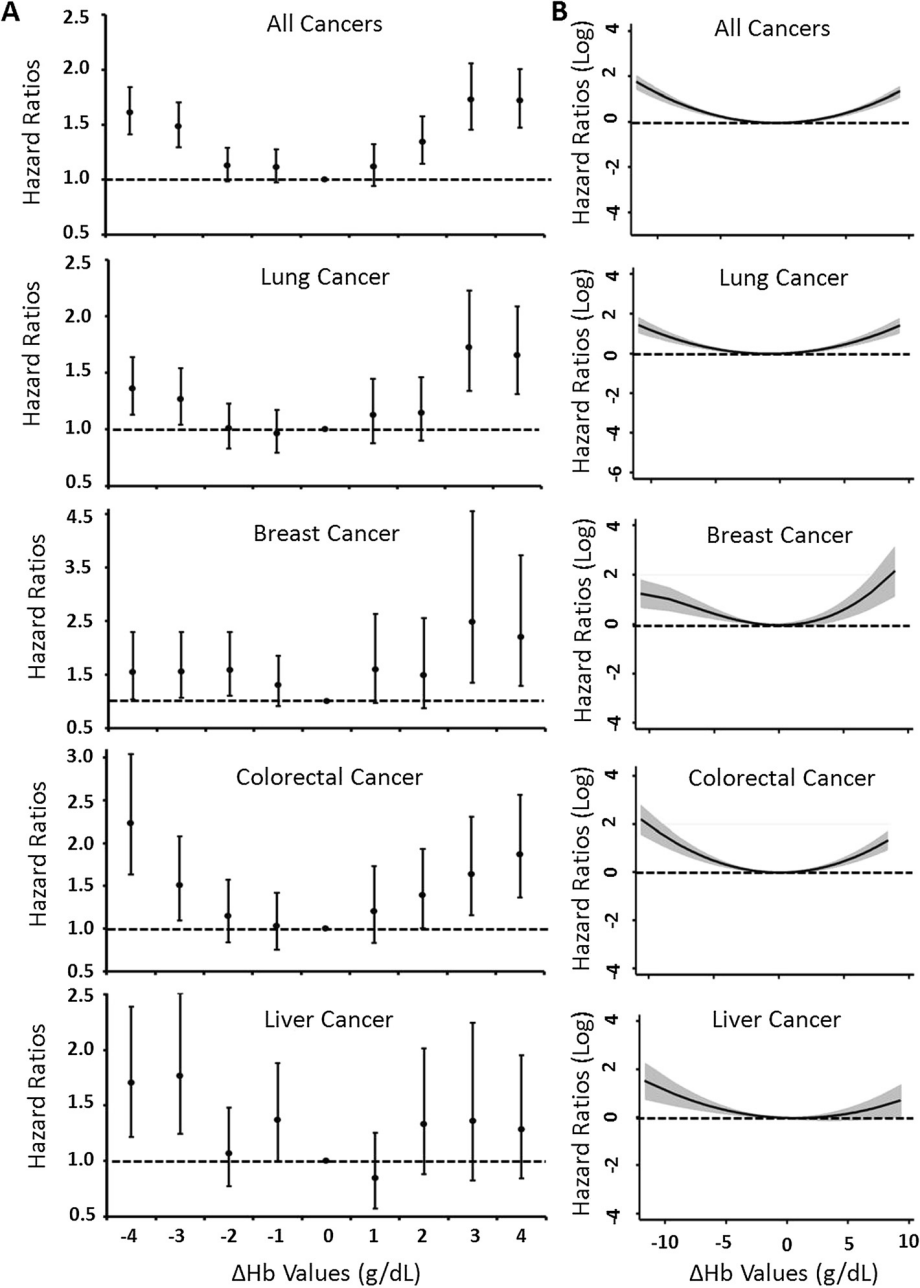
**Dose-dependent effects of Hb changes on the survival of cancer patients. (A)** Dose-dependent effects of Hb change as a categorical variable were estimated by Cox proportional hazards model by categorizing Hb changes to −4 (∆Hb<−4), -3 (−4≤∆Hb<−3), -2 (−3≤∆Hb<−2), -1 (−2≤∆Hb<−1), 1 (1<∆Hb≤2), 2 (2<∆Hb≤3), 3 (3<∆Hb≤4), 4 (∆Hb>4), and 0 (−1≤∆Hb≤1) as reference. Solid spots indicated hazards ratios and the bars indicated 95% confidence intervals. **(B)** Dose-dependent effects of Hb change as a continuous variable were analyzed by fractional polynomial regression model. Solid lines indicated hazard ratios and the shaded areas showed 95% confidence intervals. Both analyses were adjusted for age, gender, ethnicity, tumor stage, tumor grade, chemotherapy, radiation therapy and surgery. Dash lines represented the references.

To evaluate the observed associations between ∆Hb and cancer survival in a more dynamic manner, we analyzed the dose-dependent effects of ∆Hb as both a categorical and a continuous variable, using Cox proportional hazard models and fractional polynomial regression model, respectively. As shown in Figure 2, a U-shape dose-dependent effect was noticed in all cancer patients combined and in patients with individual cancers when ∆Hb was treated as a categorical (Figure 2A) or a continuous variable (Figure 2B). These data were highly consistent with that of Table 2, further demonstrating that both significantly increased and decreased Hb levels were associated with adverse survival of cancer patients.

### Time-dependent effects of Hb changes on cancer patient survival

**Figure 3:**
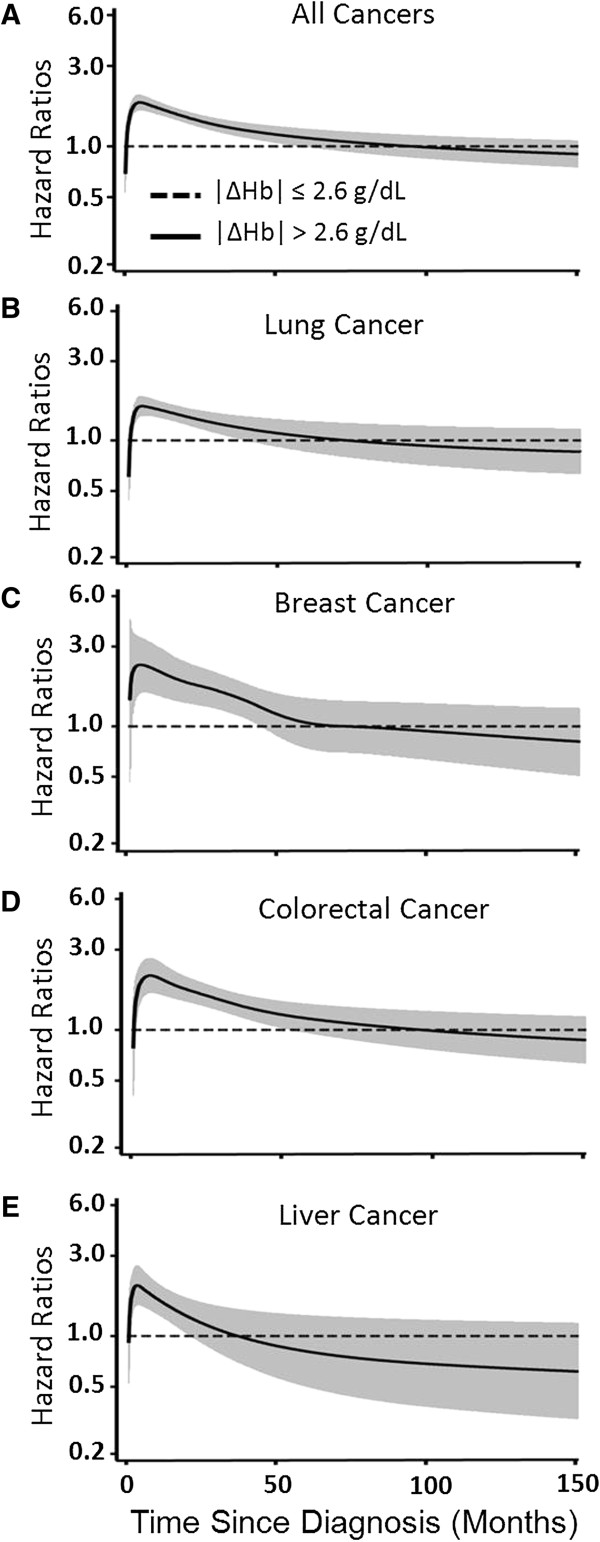
**Time-dependent effects of Hb changes on the survival of cancer patients.** Flexible parametric modeling framework was used to assess the effect by Hb changes on the overall survival of patients of **(A)** all cancers, **(B)** lung cancer, **(C)** breast cancer, **(D)** colorectal cancer, and **(E)** liver cancer. The analysis was adjusted for age, gender, ethnicity, tumor stage, tumor grade, chemotherapy, radiation therapy and surgery. Solid lines indicated hazard ratios and shaded areas showed the 95% confidence intervals. Dash lines represented the references.

We analyzed the time-dependent effects of |∆Hb| on patient survival during follow-up after diagnosis, using a flexible parametric modeling framework adjusting for host and clinical variables (Figure 3). The increased risk of death from all cancers conferred by |∆Hb| reached a peak level at 5.1 months after diagnosis, and remained significant over 54 months (Figure 3A). Similar effects were observed for individual cancers (Figures 3B to 3E).

### Death risk conferred by anemia modulated by |∆Hb|

**Figure 4:**
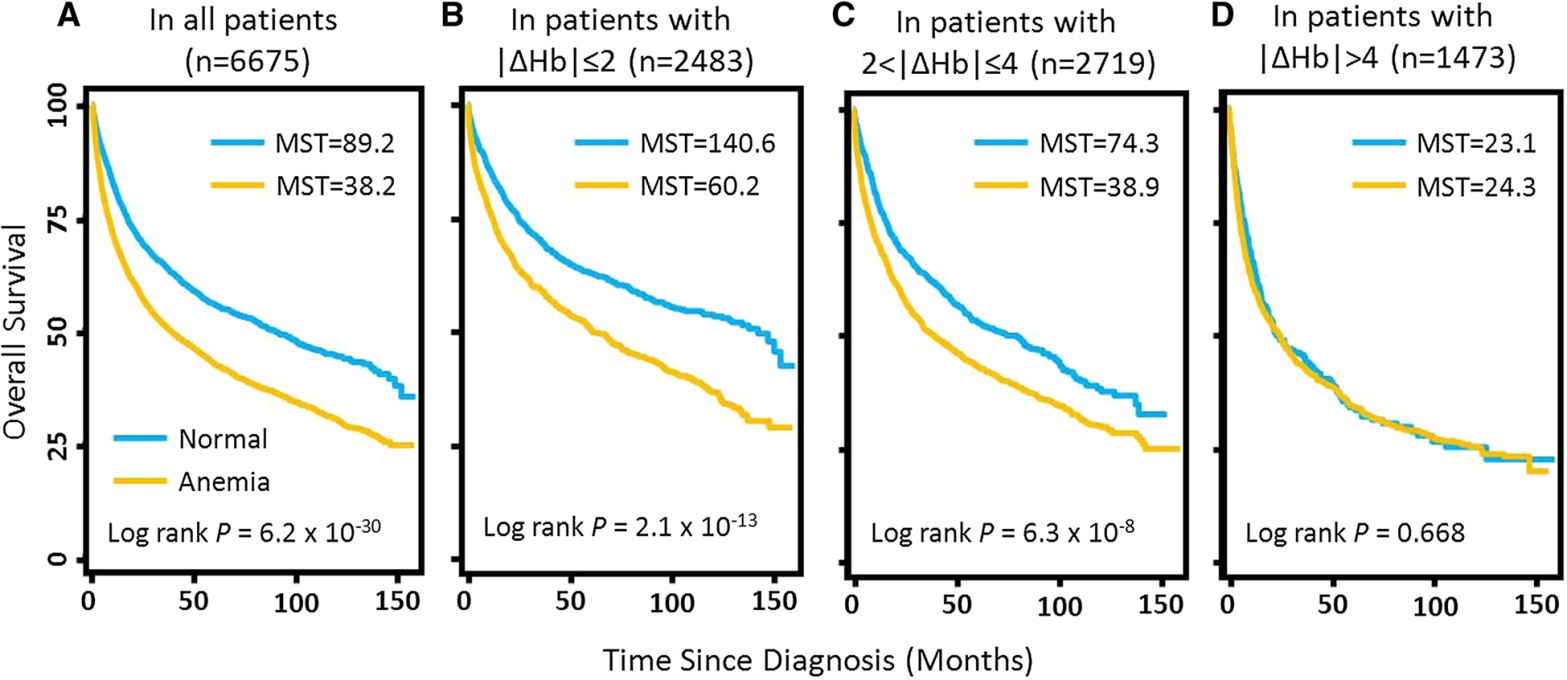
**Kaplan-Meier curves of the effects of anemia on patient survival by different levels of Hb changes.** The analysis was conducted in **(A)** all cancer patients, **(B)** patients with |∆Hb|≤ 2, **(C)** patients with 2<|∆Hb|≤ 4, and **(D)** patients with |∆Hb|> 4. Anemia patients were defined as having an average Hb level<12 g/dL. MST, median survival time.

The association between anemia and cancer mortality is influenced by many factors [8]. To evaluate if Hb changes modulate the predicative role of anemia in cancer mortality, we analyzed the association between anemia, which was indicated by the average Hb level measured within six months after diagnosis of less than 12 g/dL, and the overall survival of all cancer patients in this study stratified by different levels of |∆Hb|. In line with previous reports, Kaplan-Meier curves and log rank tests indicated that anemic patients had a much shorter survival (median survival time [MST], 38.2 months) compared with non-anemic patients (MST, 89.2 months) (*P*_log rank_=6.2 × 10^-30^) (Figure 4A). This difference remained significant in patients with small |∆Hb|. For instance, the MSTs were 140.6 months in non-anemic and 60.2 months in anemic cancer patients with |∆Hb|≤2 (*P*_log rank_=2.1 × 10^-13^) (Figure 4B). In patients with |∆Hb| between 2 and 4, the MSTs were 74.3 and 38.9 months in non-anemic and anemic patients, respectively (*P*_log rank_=6.3 × 10^-8^) (Figure 4C). However, in patients with |∆Hb|>4, no significant difference was observed between non-anemic (MST, 23.1 months) and anemic (MST, 24.3 months) patients (*P*_log rank_=0.668) (Figure 4D). The association exhibited similar patterns after adjusting for all covariates in the main effect analysis (Additional file 2: Figure S1). Moreover, similar trends were observed when the analysis was done in individual cancer sites (Additional file 2: Figure S2). Moreover, the results remained consistent when the analysis was conducted by ∆Hb instead of |∆Hb| (Additional file 2: Figure S3). These data indicated that the unfavorable survival conferred by anemia on cancer patients was modulated by ∆Hb after patient diagnosis.

## Discussion

In this study, we evaluated the association between ∆Hb measured within six months after cancer diagnosis and the overall survival of a large population of 6675 patients of four different solid tumors. Our data indicated that Hb changes after diagnosis had an adverse effect on the patient survival. The effect was in a dose-dependent manner and could persist over a long period after diagnosis. In addition, Hb changes seemed to also modulate the elevated risk of death associated with clinically defined anemia in cancer patients.

Many factors may induce anemia in cancer patients, such as bleeding, hemolysis, renal insufficiency, insufficient erythropoiesis caused by chronic inflammatory cytokines during carcinogenesis or myelosuppressive chemotherapy treatments, and nutrition deficiency [4,8,22,23]. In the current clinical setting, regardless of the different underlying causes of anemia, the evaluation of the severity of anemia mostly depends on the level of baseline Hb. However, wide variations of Hb levels among cancer patients, or even the general population, have been extensively reported, making it difficult to diagnose anemia solely based on a single measurement of Hb level at baseline. The newest updated NCCN guideline suggested that a drop of as little as 2 g/dL in Hb level, even in non-anemic patients with a high baseline Hb level, is an alarming indicator for the presence of anemia [3,24-26]. Nonetheless, as yet there has been no report systemically evaluating the role of post-diagnosis change of Hb level in predicting cancer patient survival. To the best of our knowledge, this is the first population-based study to assess the association between Hb change and overall patient survival in multiple cancers. Consistent with the suggestions from the NCCN guideline, our findings indicated that changes in Hb levels in cancer patients after diagnosis should be monitored and taken into consideration in the evaluation and determination of their treatment plans.

We noticed that both decreased and increased Hb levels were associated with a significantly poorer survival (Table 2 and Figure 2). The observation for decreased Hb was not surprising since at least a portion of Hb decrease might be accounted for by cancer- or treatment-related anemia. However, it was interesting to notice that an increase in Hb level, which usually indicates the alleviation of anemia, also conferred an increased risk of death. Moreover, in the analyses for four cancers combined, as well as individual lung cancer and breast cancer, patients with an increased Hb level showed an even more prominent adverse survival compared to those with a decreased Hb, with an HR of 1.53 versus 1.35, 1.59 versus 1.25, and 1.65 versus 1.45, respectively (Table 2). The mechanisms underlying these observations remain elusive. One potential explanation might be the use of anti-anemia medications such as ESAs or PBRC. There have been several recent studies highlighting the controversial clinical benefits and risks of using ESAs and/or PBRC to help alleviate cancer- or therapy-related anemia [9,11,12,27-30]. Some of these studies reported that overdose or over-duration in the treatment of anemia might result in elevated rate of patient death [9-12]. Our results indicated that a significant increase in Hb level after cancer diagnosis was associated with adverse patient survival, substantiating these previous reports using a population-based epidemiological approach. Nonetheless, it should be noted that although these observations are clinically plausible, they lack supports from solid clinical evidence since we currently do not have complete anemia treatment information from our chart review-derived database. Future studies with a more comprehensive collection of anemia diagnosis and treatment data are warranted to validate these hypotheses.

Clinically diagnosed anemia has been associated with unfavorable patient prognosis [31]. However, the current treatment of anemia does not always result in improved patient survival, suggesting additional criteria are needed in determining and monitoring the effects of anti-anemia therapies [32]. In the present study, we found that the significantly increased risk of death conferred by anemia was evident in those patients with a small Hb change of less than 4 g/dL, but not in those with an Hb change of more than 4 g/dL (Figure 4). These results indicated that post-diagnosis Hb changes may help improve the decision making process of anemia correction treatment in clinical settings. Specifically, it raised the question whether anti-anemia medications should continuously be administered to patients with a relatively large change in Hb level in a short period of time. Nonetheless, since our analysis did not include anti-anemia treatments which could significantly confound the results, independent studies with more complete anti-anemia treatment data are needed to validate these findings with regard to their clinical relevance.

Although anemia is a ubiquitous comorbidity in cancer patients, its prevalence in different cancer types vary widely, ranging from 30% to 90% [1]. A recent large population-based study reported that CRC patients had the highest incidence of anemia at diagnosis among all solid tumors, whereas there was no apparent Hb change in CRC patients within five years before diagnosis [33]. In the present study, post-diagnosis Hb changes in CRC patients were most significantly associated with overall survival among the four cancer types, as evidenced by the 100% validations with *P*<0.01 in bootstrap resampling in the analyses of both |∆Hb| and ∆Hb levels (Table 2). In comparison, for breast cancer, no significant results were validated for the association between ∆Hb and patient survival. It remains to be determined as to whether gender played a role in these observations. We compared the |∆Hb| of breast cancer patients to that of female patients of the other three cancers and found that breast cancer patients had the smallest Hb changes among all female cancer patients (*P*<0.001 for all three comparisons, Figure 1). These differences were likely due to cancer type instead of gender status, because such significant differences were not identified between male and female patients in the other three cancers (*P*= 0.056, 0.096 and 0.850 for lung, colorectal and liver cancers, respectively, Figure 1). Although the mechanism underlying these cancer type-specific findings remains to be investigated, these data suggest that the use of Hb changes in anemia management and patient prognostication should be individually assessed for different cancer types.

There are several strengths in this study. We had a large population of 6675 patients from a single institute and our conclusions were consistent among four different cancer types. The study was focused on the extensive analysis of a single variable and, thus, did not have the multiple comparison issue. The findings were highly statistically significant in both the Cox regression and the log rank analyses with strict internal validations using bootstrap resampling, indicating that the possibility of false positive findings is unlikely. Meanwhile, our study also has limitations. Because this study used archived clinical data obtained from chart review instead of prospectively collected data, many cancer type-specific data did not have complete and/or standardized records in medical charts and thus had a relatively large percentage of missing values. In addition, important confounding variables such as anti-anemia modalities and treatment toxicities were not complete in our database and thus not adjusted in the multivariate analyses. Therefore, our data, although highly statistically significant and biologically plausible, need to be further substantiated in more rigorous studies using large independent and prospective populations with a more comprehensive collection of relevant confounding variables.

## Conclusions

In summary, our findings suggested that post-diagnosis Hb changes, regardless of the baseline Hb levels and the direction of changes, associate with the overall survival of the patients of various cancers and should be taken into consideration in the tailored correction of anemia treatment. Since cancer- or treatment-related anemia is present in up to 90% of patients, our finding has considerable significance at the population level. Moreover, because Hb level is frequently measured in the routinely tested complete blood count panel, Hb changes may be a potentially important variable that can be incorporated with other host and clinical factors to build cancer prognosis assessment models.

## Competing interests

The authors declare that they have no competing interests

## Authors’ contribution

SW conducted data collection, study design and manuscript writing. YL conducted data analysis. RM contributed to study design and manuscript writing. BL conducted data analysis and manuscript writing. JP contributed to study design and manuscript writing. AB contributed to study design and manuscript writing. GC contributed to study design, data analysis, and manuscript writing. JX contributed to study design and manuscript writing. HY conducted data collection, study design, data analysis, and manuscript writing. All authors read and approved the final manuscript.

## Pre-publication history

The pre-publication history for this paper can be accessed here:

http://www.biomedcentral.com/1471-2407/13/340/prepub

## Supplementary Material

Additional file 1: Table S1 The association between baseline Hb level and cancer survival. **Table S2.** Joint effect of baseline Hb level and Hb change on cancer overall survival. Click here for file

Additional file 2: Figure S1 Kaplan-Meier curves of the effects of anemia on patient survival by different levels of Hb changes adjusting for all covariates. The analysis was conducted in (A) all cancer patients, (B) patients with |∆Hb|≤ 2, (C) patients with 2<|∆Hb|≤ 4, and (D) patients with |∆Hb|> 4. Anemia patients were defined as having an average Hb level<12 g/dL. The analyses were adjusting for age, gender, ethnicity, tumor stage, tumor grade, chemotherapy, radiation therapy and surgery. **Figure S2.** Kaplan-Meier curves of the effects of anemia on patient survival by different levels of Hb changes in individual cancer site. The analysis was conducted in (A) Lung cancer, (B) Breast cancer, (C) Colorectal cancer, and (D) Liver cancer. Anemia patients were defined as having an average Hb level<12 g/dL. **Figure S3.** Kaplan-Meier curves of the effects of anemia on patient survival by actual changes of Hb level. The analysis was conducted in (A) patients with -2 ≤ ∆Hb < 0, (B) patients with -4 ≤ ∆Hb < 2, (C) patients with ∆Hb < -4, (D) patients with 0 ≤ ∆Hb ≤ 2, (E) patients with 2< ∆Hb ≤ 4, and (F) patients with ∆Hb > 4. Anemia patients were defined as having an average Hb level<12 g/dL. Click here for file
