# Supplementary material for: Post-diagnosis hemoglobin change associates with overall survival of multiple malignancies – results from a 14-year hospital-based cohort of lung, breast, colorectal, and liver cancers
Source: BMC Cancer. 2013 Jul 10;13:340. doi: 10.1186/1471-2407-13-340 (PMC3710492; doi:10.1186/1471-2407-13-340)
Supplement: Additional file 1: Table S1 — The association between baseline Hb level and cancer survival. Table S2. Joint effect of baseline Hb level and Hb change on cancer overall survival. [file 1471-2407-13-340-S1.docx]

| **Table S1. The association between baseline Hb level and cancer survival** | | | | | |
| --- | --- | --- | --- | --- | --- |
| Caner types | Baseline Hb values | Dead | Alive | HR (95% CI)* | *P* value |
| All cancers |  |  |  |  |  |
|  | Hb ≥ 12 | 1571 | 1782 | 1.00 |  |
|  | Hb < 12 | 1971 | 1351 | 1.33 (1.24-1.43) | 4.7 x 10 ^-16^ |
| Lung cancer |  |  |  |  |  |
|  | Hb ≥ 12 | 924 | 355 | 1.00 |  |
|  | Hb < 12 | 871 | 217 | 1.20 (1.09-1.32) | 1.6 x 10 ^-4^ |
| Breast cancer |  |  |  |  |  |
|  | Hb ≥ 12 | 179 | 886 | 1.00 |  |
|  | Hb < 12 | 186 | 488 | 1.53 (1.23-1.89) | 1.1 x 10 ^-4^ |
| Colorectal cancer | |  |  |  |  |
|  | Hb ≥ 12 | 213 | 402 | 1.00 |  |
|  | Hb < 12 | 661 | 584 | 1.58 (1.35-1.84) | 1.5 x 10 ^-8^ |
| Liver cancer |  |  |  |  |  |
|  | Hb ≥ 12 | 255 | 139 | 1.00 |  |
|  | Hb < 12 | 253 | 62 | 1.53 (1.27-1.83) | 4.6 x 10 ^-6^ |
| *Adjusted for age, gender, ethnicity, tumor stage, tumor grade, chemotherapy, radiation therapy, and surgery. | | | | | |

| **Table S2. Joint effect of baseline Hb level and Hb change on cancer overall survival** | | | | | | |
| --- | --- | --- | --- | --- | --- | --- |
| Cancer types | Baseline Hb value | Hb changes | Dead | Alive | HR (95% CI)* | *P* value |
| All cancers |  |  |  |  |  |  |
|  | Hb ≥ 12 | \|∆Hb\| ≤ 2 | 477 | 772 | 1.00 |  |
|  | Hb ≥ 12 | \|∆Hb\| > 2 | 594 | 559 | 1.23 (1.09-1.39) | 0.0008 |
|  | Hb < 12 | \|∆Hb\| ≤ 2 | 646 | 585 | 1.34 (1.19-1.51) | 1.6 x 10 ^-6^ |
|  | Hb < 12 | \|∆Hb\| > 2 | 1825 | 1217 | 1.75 (1.58-1.94) | 4.9 x 10 ^-26^ |
| Lung cancer |  |  |  |  |  |  |
|  | Hb ≥ 12 | \|∆Hb\| ≤ 2 | 234 | 89 | 1.00 |  |
|  | Hb ≥ 12 | \|∆Hb\| > 2 | 343 | 121 | 1.11 (0.94-1.32) | 0.2098 |
|  | Hb < 12 | \|∆Hb\| ≤ 2 | 297 | 100 | 1.16 (0.98-1.38) | 0.0852 |
|  | Hb < 12 | \|∆Hb\| > 2 | 921 | 262 | 1.52 (1.31-1.76) | 3.1 x 10 ^-8^ |
| Breast cancer |  |  |  |  |  |  |
|  | Hb ≥ 12 | \|∆Hb\| ≤ 2 | 74 | 469 | 1.00 |  |
|  | Hb ≥ 12 | \|∆Hb\| > 2 | 47 | 177 | 1.27 (0.86-1.88) | 0.2365 |
|  | Hb < 12 | \|∆Hb\| ≤ 2 | 81 | 288 | 1.44 (1.04-1.99) | 0.0263 |
|  | Hb < 12 | \|∆Hb\| > 2 | 163 | 440 | 1.75 (1.30-2.35) | 0.0002 |
| Colorectal cancer | |  |  |  |  |  |
|  | Hb ≥ 12 | \|∆Hb\| ≤ 2 | 66 | 149 | 1.00 |  |
|  | Hb ≥ 12 | \|∆Hb\| > 2 | 110 | 213 | 1.22 (0.90-1.67) | 0.1980 |
|  | Hb < 12 | \|∆Hb\| ≤ 2 | 164 | 179 | 1.52 (1.14-2.03) | 0.0046 |
|  | Hb < 12 | \|∆Hb\| > 2 | 534 | 445 | 2.27 (1.75-2.94) | 6.2 x 10 ^-10^ |
| Liver cancer |  |  |  |  |  |  |
|  | Hb ≥ 12 | \|∆Hb\| ≤ 2 | 103 | 65 | 1.00 |  |
|  | Hb ≥ 12 | \|∆Hb\| > 2 | 94 | 48 | 1.35 (1.01-1.80) | 0.0437 |
|  | Hb < 12 | \|∆Hb\| ≤ 2 | 104 | 18 | 1.72 (1.30-2.27) | 0.0002 |
|  | Hb < 12 | \|∆Hb\| > 2 | 207 | 70 | 1.82 (1.43-2.32) | 1.4 x 10 ^-6^ |
| *Adjusted for age, gender, ethnicity, tumor stage, tumor grade, chemotherapy, radiation therapy, and surgery. | | | | | | |
