# Supplementary material for: Post-diagnosis hemoglobin change associates with overall survival of multiple malignancies – results from a 14-year hospital-based cohort of lung, breast, colorectal, and liver cancers
Source: BMC Cancer. 2013 Jul 10;13:340. doi: 10.1186/1471-2407-13-340 (PMC3710492; doi:10.1186/1471-2407-13-340)

**Supplementary Figure Legends:**

**Figure S1. Kaplan-Meier curves of the effects of anemia on patient survival by different levels of Hb changes adjusting for all covariates.** The analysis was conducted in (**A**) all cancer patients, (**B**) patients with |∆Hb|≤ 2, (**C**) patients with 2<|∆Hb|≤ 4, and (**D**) patients with |∆Hb|> 4. Anemia patients were defined as having an average Hb level<12 g/dL. The analyses were adjusting for age, gender, ethnicity, tumor stage, tumor grade, chemotherapy, radiation therapy and surgery.

**Figure S2. Kaplan-Meier curves of the effects of anemia on patient survival by different levels of Hb changes in individual cancer site.** The analysis was conducted in (**A**) Lung cancer, (**B**) Breast cancer, (**C**) Colorectal cancer, and (**D**) Liver cancer. Anemia patients were defined as having an average Hb level<12 g/dL.

**Figure S3. Kaplan-Meier curves of the effects of anemia on patient survival by actual changes of Hb level.** The analysis was conducted in (**A**) patients with -2 ≤ ∆Hb < 0, (**B**) patients with -4 ≤ ∆Hb < 2, (**C**) patients with ∆Hb < -4, (**D**) patients with 0 ≤ ∆Hb ≤ 2, (**E**) patients with 2< ∆Hb ≤ 4, and (**F**) patients with ∆Hb > 4. Anemia patients were defined as having an average Hb level<12 g/dL.

**
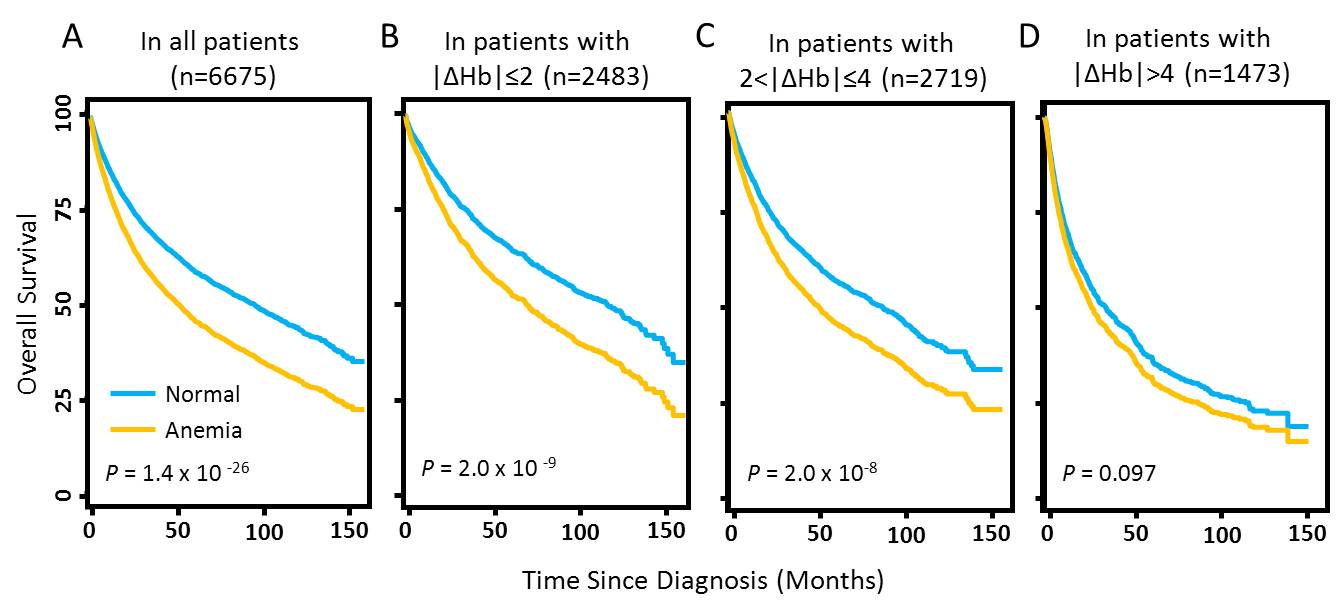
Figure S1**.

**Figure S2.**


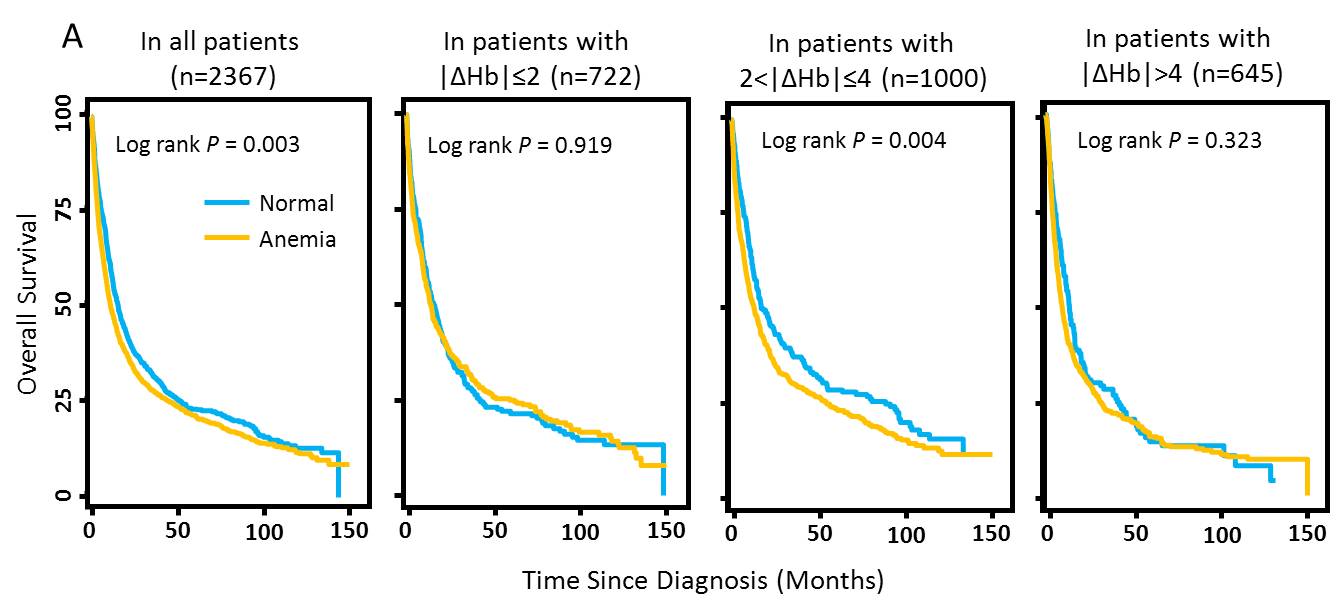


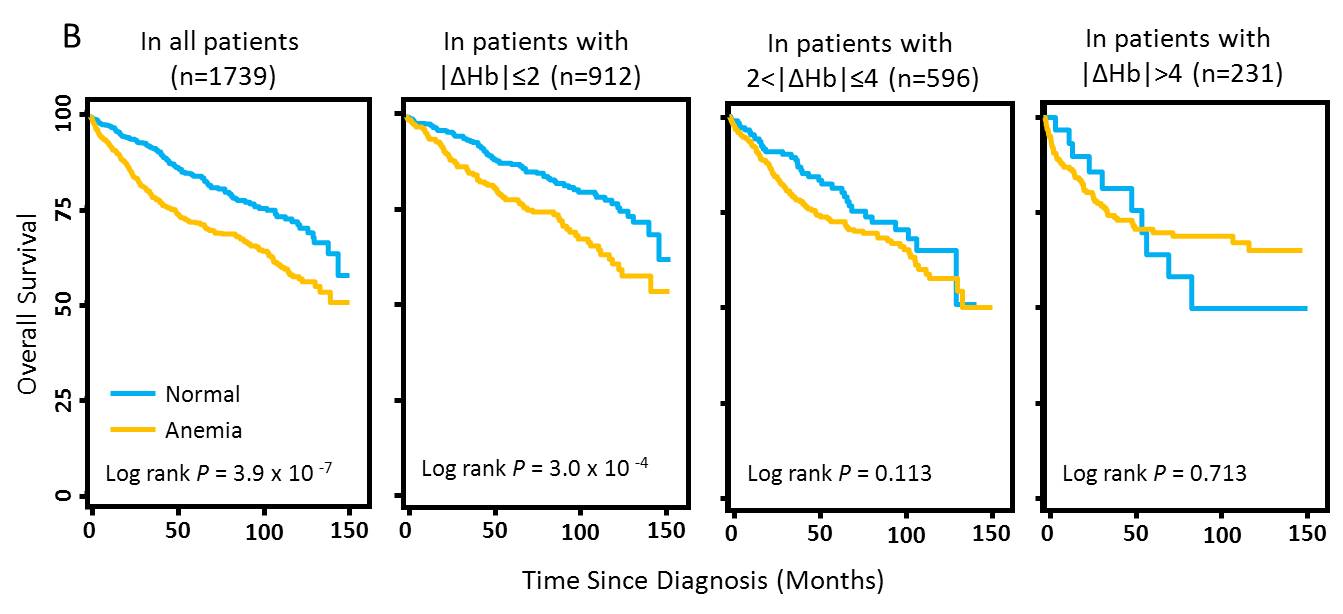

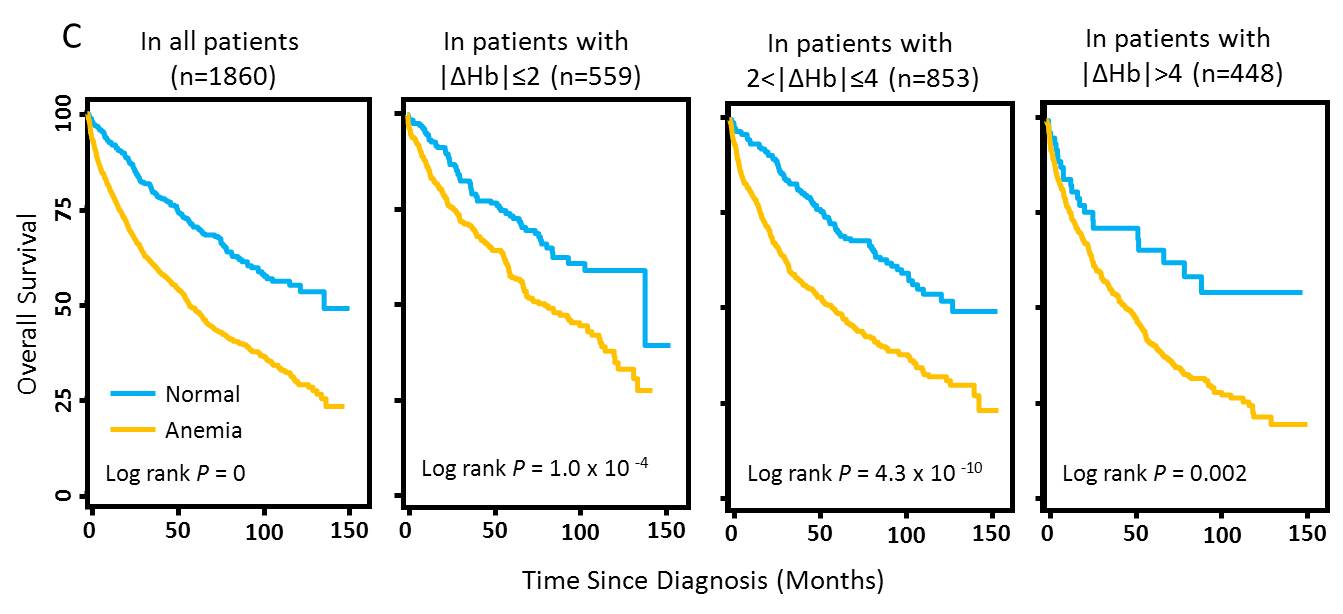


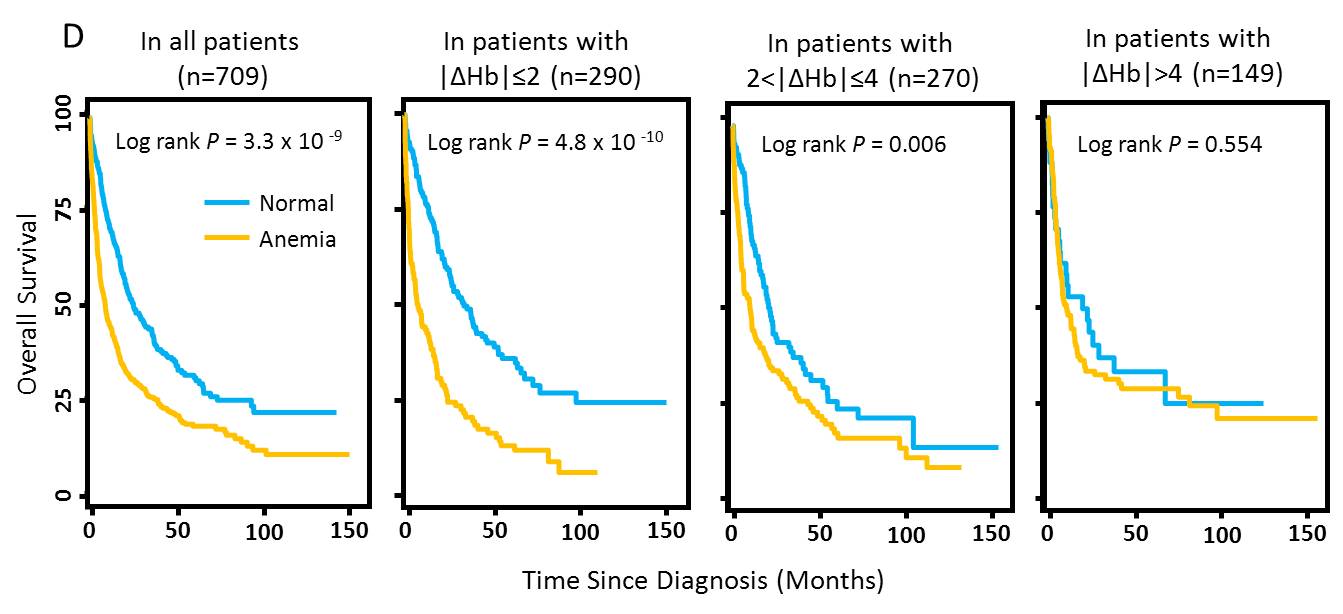


**Figure S3.**


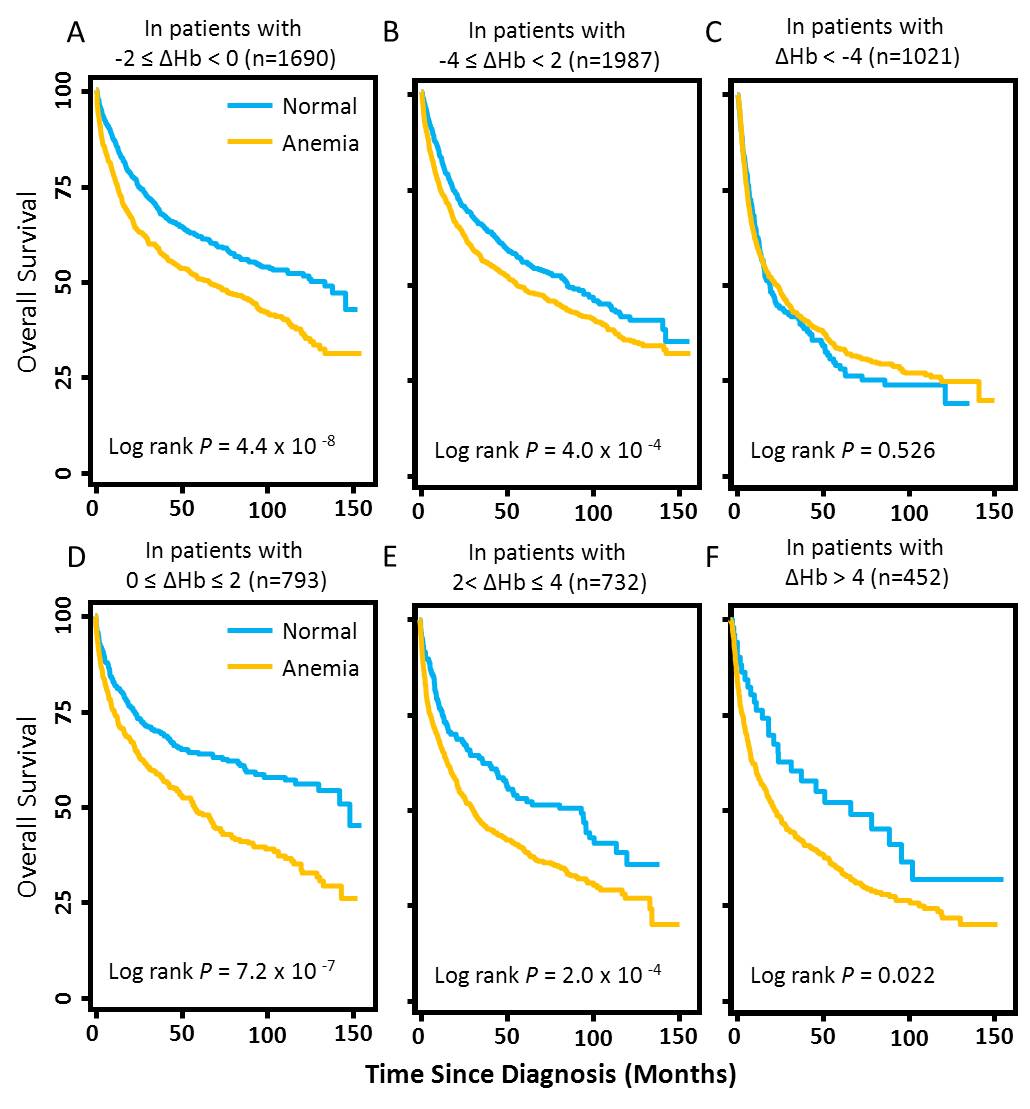

Supplement: Additional file 2: Figure S1 — Kaplan-Meier curves of the effects of anemia on patient survival by different levels of Hb changes adjusting for all covariates. The analysis was conducted in (A) all cancer patients, (B) patients with |∆Hb|≤ 2, (C) patients with 2<|∆Hb|≤ 4, and (D) patients with |∆Hb|> 4. Anemia patients were defined as having an average Hb level<12 g/dL. The analyses were adjusting for age, gender, ethnicity, tumor stage, tumor grade, chemotherapy, radiation therapy and surgery. Figure S2. Kaplan-Meier curves of the effects of anemia on patient survival by different levels of Hb changes in individual cancer site. The analysis was conducted in (A) Lung cancer, (B) Breast cancer, (C) Colorectal cancer, and (D) Liver cancer. Anemia patients were defined as having an average Hb level<12 g/dL. Figure S3. Kaplan-Meier curves of the effects of anemia on patient survival by actual changes of Hb level. The analysis was conducted in (A) patients with -2 ≤ ∆Hb < 0, (B) patients with -4 ≤ ∆Hb < 2, (C) patients with ∆Hb < -4, (D) patients with 0 ≤ ∆Hb ≤ 2, (E) patients with 2< ∆Hb ≤ 4, and (F) patients with ∆Hb > 4. Anemia patients were defined as having an average Hb level<12 g/dL. [file 1471-2407-13-340-S2.docx]
